# A Systematic Review and Meta-Analysis of Qigong for the Fibromyalgia Syndrome

**DOI:** 10.1155/2013/635182

**Published:** 2013-10-31

**Authors:** Romy Lauche, Holger Cramer, Winfried Häuser, Gustav Dobos, Jost Langhorst

**Affiliations:** ^1^Department of Internal and Integrative Medicine, Kliniken Essen-Mitte, Faculty of Medicine, University of Duisburg-Essen, 45276 Essen, Germany; ^2^Department Internal Medicine I, Klinikum Saarbrücken, 66119 Saarbrücken, Germany; ^3^Department of Psychomsomatic, Medicine and Psychotherapy, Technische Universität München, 81865 München, Germany

## Abstract

*Objectives*. The fibromyalgia syndrome (FMS) is a chronic condition with only few evidence-based complementary and alternative therapies available. This paper presents a systematic review and meta-analysis of the effectiveness of Qigong for fibromyalgia syndrome. *Methods*. The PubMed/MEDLINE, Cochrane Library, Embase, PsycINFO, and Cambase databases were screened in December 2012 to identify randomized controlled trials comparing Qigong to control interventions. Major outcome measures were pain and quality of life; and secondary outcomes included sleep quality, fatigue, depression, and safety. Standardized mean differences (SMD) and 95% confidence intervals were calculated. *Results*. Seven trials were located with a total of 395 FMS patients. Analyses revealed low quality evidence for short-term improvement of pain, quality of life, and sleep quality and very low quality evidence for improvement of fatigue after Qigong for FMS, when compared to usual care. No evidence was found for superiority of Qigong compared to active treatments. No serious adverse events were reported. *Discussion*. This systematic review found that Qigong may be a useful approach for FMS patients. According to the quality of evidence, only a weak recommendation for Qigong can be made at this point. Further high quality RCTs are required for the conclusive judgment of its long-term effects.

## 1. Introduction

The fibromyalgia syndrome (FMS) is a chronic condition characterized by chronic widespread pain, fatigue, cognitive disturbances, sleep disorders, and a high amount of somatic and psychological distress [1, 2]. The prevalence of FMS in the general population has been estimated between 2.9% and 3.8% in Europe [3, 4] with women being more frequently affected than men [1]. Due to lack of data, only a few complementary therapies can be recommended. One such recommendation includes the application of meditative exercise techniques such as yoga, Qigong, or tai chi [5]. The most recent consumers report in Germany stated that 18.4% of fibromyalgia patients currently used some kind of meditative exercises, including Qigong [6]. Despite its recommendation, no conclusive judgment on Qigong's efficacy has been drawn by reviews [7–9].

Qigong is a Chinese medical exercise that combines static or dynamic physical exercises, breathing exercises, and meditation [10]. Qigong aims to increase the energy flow, the so-called qi, through the body [11]. Qigong is well accepted in western societies; for example, in the US estimated 500,000 individuals used Qigong for coping with diseases such as musculoskeletal conditions, severe sprains, and asthma [12], or even with cancer [13]. Qigong has proved to be effective for physical conditions and for psychological well-being [10], which could make it a valuable treatment option for fibromyalgia patients, who suffer from physical and psychological complaints.

Prior reviews have been conducted to test the efficacy of Qigong for FMS [7–9]; however, no reliable conclusions could be drawn due to the small number of included trials. The aim of this systematic review and meta-analysis was to assess short- and long-term efficacy and safety of Qigong in patients with FMS compared to control interventions. 

## 2. Materials and Methods

### 2.1. Protocol and Registration

This review was planned and conducted in accordance with the Preferred Reporting Items for Systematic Reviews and Meta-Analyses guidelines (PRISMA) [14], the recommendations of the Cochrane Musculoskeletal Group [15, 16] Grading of Recommendations Assessment, Development and Evaluation and the recommendations (GRADE) [17]. The protocol was not registered on any database.

### 2.2. Eligibility Criteria

To be eligible for review, studies were required to meet the following conditions.

1. *Types of study designs:* randomized controlled trials (RCTs) were eligible.
2. *Types of participants:* studies of patients with fibromyalgia were eligible, regardless of age, condition's duration, or intensity. No further restriction regarding diagnostic procedures was applied.
3. *Types of interventions:* studies that compared Qigong with no treatment, usual care, or any active treatment were eligible. No restrictions were applied to the details of the Qigong. Cointerventions were allowed, but studies with cointerventions were excluded in the subsequent sensitivity analyses.
4. *Types of outcomes:* studies were eligible if they assessed at least one major patient-centered outcome, namely, pain or quality of life. Secondary outcomes were sleep quality, fatigue, depression, and safety.
5. *Length of followup:* no restrictions regarding length of followup were applied. Short-term effects were defined as measures taken directly after the intervention and long-term effects as measures taken closest to 12 months after randomization.
6. *Accessibility of data:* studies were eligible only if they were published as full papers. No language restriction was applied.

### 2.3. Literature Search

The following electronic databases were searched from their inception through to December 31, 2012: PubMed/MEDLINE, Embase, PsycINFO, the Cochrane Library, and Cambase. The literature search, which was constructed around search terms for “Qigong” and “fibromyalgia syndrome,” was adapted for each database as necessary. For example, the following search strategy was used on the PubMed/MEDLINE database: (Fibromyalgia [MESH] OR fibromyalgia [Title/Abstract] OR fibrositis [Title/Abstract] OR widespread pain [Title/Abstract]) AND (Qigong [MESH] OR Qigong [Title/Abstract] OR Chi gong [Title/Abstract] OR Chi kung [Title/Abstract] OR breathing exercises [Title/Abstract]). The reference lists of identified original articles or reviews were also searched manually for relevant articles. 

### 2.4. Study Selection

At first, all duplicate papers were removed. Two reviewers then screened the abstracts of the remaining papers individually. They went on to obtain the full papers for all potentially eligible studies. The studies were then checked for eligibility, with eligible papers being included in the systematic review. Papers that provided data on relevant clinical outcomes as defined in the next section were also included in the meta-analysis.

### 2.5. Data Collection

Two reviewers independently extracted data on studies' characteristics (participants, interventions, control conditions, cointerventions, outcome measures, and results). Disagreements were discussed with a third reviewer and resolved by agreement. If data could not be extracted from the original published papers, their authors were contacted.

#### 2.5.1. Outcome Measures

To be eligible, studies had to at least measure one major outcome, namely:

1. pain intensity, measured on a visual analogue scale (VAS), a numerical rating scale (NRS), the pain subscale of the Fibromyalgia Impact Questionnaire (FIQ) [18], or on another validated specific measure;
2. disease specific health-related quality of life, assessed by the Fibromyalgia Impact Questionnaire (FIQ) [18] or any other validated instrument;
3. generic health-related quality of life, assessed by the World Health Organization Quality of Life Questionnaire (WHOL-QOL) [19], the Short Form 36 Health Survey Questionnaire (SF-36) [20], or on another validated instrument eligible for patients with fibromyalgia. In case of multidimensional instruments with several component summaries, only the physical score was used for analyses.

Secondary outcomes included the following.

1. Sleep quality was assessed on a visual analogue scale (VAS), a numerical rating scale (NRS), the Pittsburgh Sleep Quality Index (PSQI) [21], or on another validated sleep quality index. If studies used multiple instruments, the PSQI was preferred over the VAS.
2. Fatigue was measured on a visual analogue scale (VAS), a numerical rating scale (NRS), the Multidimensional Fatigue Inventory (MFI) [22], or on another validated fatigue questionnaire. If studies used multiple instruments, the MFI was preferred over the VAS.
3. Depression was included where this was measured on the Beck Depression Inventory (BDI) [23] or on another validated depression inventory. If studies used multiple instruments, the BDI was preferred over the others.
4. Patients' safety was defined as any adverse event occurring during a study.

#### 2.5.2. Risk of Bias in Individual Studies

The risk of bias at study level was assessed by two independent reviewers using the 2006 Method guidelines for systematic reviews of the Cochrane Musculoskeletal Group [16]. These guidelines recommend the imposition of seven quality criteria, each of which is rated as “low risk,” “high risk,” or “unclear risk of bias.” These criteria relate to the following risk of bias categories: random sequence generation (selection bias), allocation concealment (selection bias), blinding of participants and personnel (performance bias), blinding of outcome assessors (detection bias), incomplete outcome data (attrition bias), selective reporting (reporting bias), and other bias relating to major study flaws. The risk of bias within each domain was used to perform sensitivity analyses.

### 2.6. Data Analysis

Studies were analyzed separately for their type of intervention (waitlist/usual care versus active treatments) and for short- and long-term effects. Short-term outcomes were defined as those from measures applied directly after treatment and long-term outcomes from measures applied closest to the six months after randomization.

#### 2.6.1. Assessment of Effect Size

If at least two studies presented data on an outcome, then meta-analysis was undertaken using Review Manager 5 software (version 5.2, The Nordic Cochrane Centre, Copenhagen, Denmark).

Standardized mean differences (SMD) with 95% confidence intervals (CI) were calculated as the mean group differences divided by the respective pooled standard deviations [24]. Where no standard deviations were available, standard errors, confidence intervals, or *t* values were used to calculate them. A random effect model was applied.

The magnitude of the overall effect size was classified according to Cohen's categories: a small effect size was defined as a SMD = 0.2 to 0.5, moderate effect size: SMD = 0.5 to 0.8, and large effect size: SMD > 0.8 [25].

A negative standardized mean difference was defined to indicate the beneficial effects of Qigong, as compared to the control interventions, for all outcomes except generic quality of life, where a positive SMD corresponded to enhanced well-being. If necessary, patients' scores were inverted and the mean score was multiplied by −1.

#### 2.6.2. Assessment of Heterogeneity

Statistical heterogeneity between the reviewed studies was quantified by determination of *I* ^2^. *I* ^2^ > 30%, *I* ^2^ > 50%, and *I* ^2^ > 75% were defined to indicate moderate, substantial, and considerable heterogeneity, respectively [15]. A *P* value ≤0.10 from the *χ* ^2^ test was taken to indicate significant heterogeneity [15].

#### 2.6.3. Subgroup and Sensitivity Analyses

Subgroup analyses were conducted for studies that applied Qigong as part of a comprehensive program versus studies that used Qigong as a standalone treatment.

Sensitivity analyses to test the robustness of any significant results were conducted by comparing the results of studies with high risk versus low risk at the domains selection bias, detection bias, attrition bias, and other risks. If statistical heterogeneity was present in the respective meta-analysis, sensitivity analyses were conducted by subsequent exclusion of single studies.

#### 2.6.4. Risk of Bias across Studies

If at least ten studies were included in a meta-analysis, the risk of publication bias was assessed by visual analysis of funnel plots generated by Review Manager 5.1 software. Roughly symmetrical funnel plots indicate a low risk of publication bias, while asymmetrical funnel plots indicate a high risk of such bias [26].

#### 2.6.5. Quality of Evidence

The quality of evidence for each outcome was judged according to the GRADE recommendations [17] based on the methodological quality and the confidence in the results of the meta-analysis.

1. High quality: further research is very unlikely to change the confidence in the estimate of effect.
2. Moderate quality: further research is likely to have an important impact on the confidence in the estimate of effect and may change the estimate.
3. Low quality: further research is very likely to have an important impact on our confidence in the estimate of effect and is likely to change the estimate.
4. Very low quality: any estimate of effect is very uncertain.

#### 2.6.6. Strength of Recommendation

The strength of recommendation for Qigong as a therapeutic option is judged according to GRADE with either “strong” or “weak” [17]. This recommendation takes into account the quality of evidence and the risk of undesirable effects.

## 3. Results

### 3.1. Study Selection

**Figure 1:**
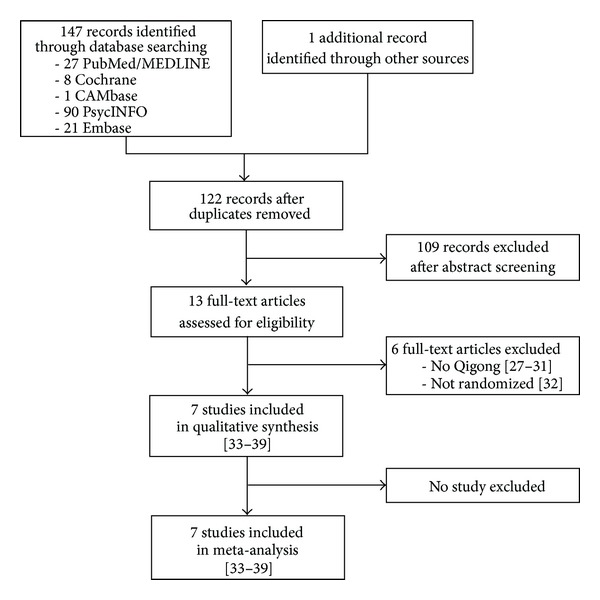
Flow chart of results of the literature search.

The literature search retrieved 147 records, of which 26 were duplicates (Figure 1). After abstract screening, 103 records were excluded. Of the remaining 13 articles that were assessed as full text, 6 either referred to studies that did not investigate Qigong (*N* = 5) [27–31] or that were not randomized (*N* = 1) [32]. At the end, 7 studies with a total of 395 patients could be included in the qualitative and quantitative analysis [33–39].

### 3.2. Study Characteristics

The characteristics of the study samples, interventions, outcome measures, and results are shown in Table 1.

#### 3.2.1. Setting and Participant Characteristics

Trials originated from Australia [38], Canada [36], Sweden [34, 39], Italy [37], and USA [33, 35]. All except for one study included patients who had been diagnosed according to the American College of Rheumatology (ACR) diagnostic criteria [33, 35–39], and one study did not refer to the diagnostic procedure [34]. Four studies included adults of both genders [33, 35–37], whereas three studies included only females [34, 39] or children [38]. All studies were conducted in primary or secondary care settings. 

#### 3.2.2. Intervention Characteristics

Qigong was practiced between 6 and 12 weeks with one or two supervised sessions a week and additional home practices up to two sessions a week. In one study [36] patients practiced Qigong at home until the 6-month followup.

The studies' control interventions differed widely. Three studies used a wait-list or usual care group [34, 36, 39], one study included a sham Qigong [35], one study used an education and support group [33], one study used a body awareness training (Rességuier method) [37], and another one aerobic exercises [38]. 

#### 3.2.3. Outcome Measures

Pain was assessed as an outcome measure in all studies, with three studies using the NRS [34, 36, 37] and one using VAS [38], the pain scale of the FIQ [39], a myalgic score based on the tender point sensitivity [33], or the Short-Form McGill Pain Questionnaire (SMPQ) [35]. Disease specific quality of life was measured in five studies using the FIQ [33, 35–37, 39] and in one study using the Childhood Health Assessment Questionnaire (C-HAQ) [38]. Generic quality of life was assessed in four studies by means of the WHO-QOL [34], the SF-36 [36, 37], or the Quality of My Life Scale (QOML) [38].

Sleep quality was assessed in four studies [34–37] with two studies using the PSQI [35, 36] and two studies using a NRS [34, 37]. Fatigue was measured in one study using a NRS [34], the MFI [35], the fatigue scale of the FIQ [39], or the fatigue module of the Pediatric Quality of life Inventory (PedsQL) [38]. Depression was measured in two studies using the BDI [33, 34], in one study using the subscale of the FIQ [39], the subscale depression of the Hamilton Anxiety and Depression Scale (HADS) [37], and the Childhood Depression Inventory (CDI) [38]. Safety was assessed and reported in two trials [36, 38]. 

Short-term effects were assessed in all studies, but only two studies investigated long-term effects [33, 39]. The study of Maddali Bongi et al. [37] investigated long-term effect; however, due to the cross-over character of the study the follow-up data could not be used.

#### 3.2.4. Risk of Bias in Individual Studies

No study was considered to be serious flawed (see Table 2). Studies' risk of selection bias was mixed, with four out of seven studies having low risk at random sequence generation [33, 36–38] and only three studies having low risk at allocation concealment [33, 37, 38]. All other studies have unclear risk of selection bias since they did not describe the procedures in detail.

The risk of performance bias was mostly unclear and one study had high risk of bias in that domain [35]. Detection bias was low in all studies. Attrition bias was mixed with three out of seven studies having a high risk of bias, mainly because of high drop-out rates or nondescribing reasons for withdrawals [33, 38, 39]. Reporting bias was only high risk in one study [34].

### 3.3. Qigong versus Waitlist/Usual Care

#### 3.3.1. Analyses of Effects of Qigong versus Waitlist/Usual Care

*Major Outcomes*

**Figure 2:**
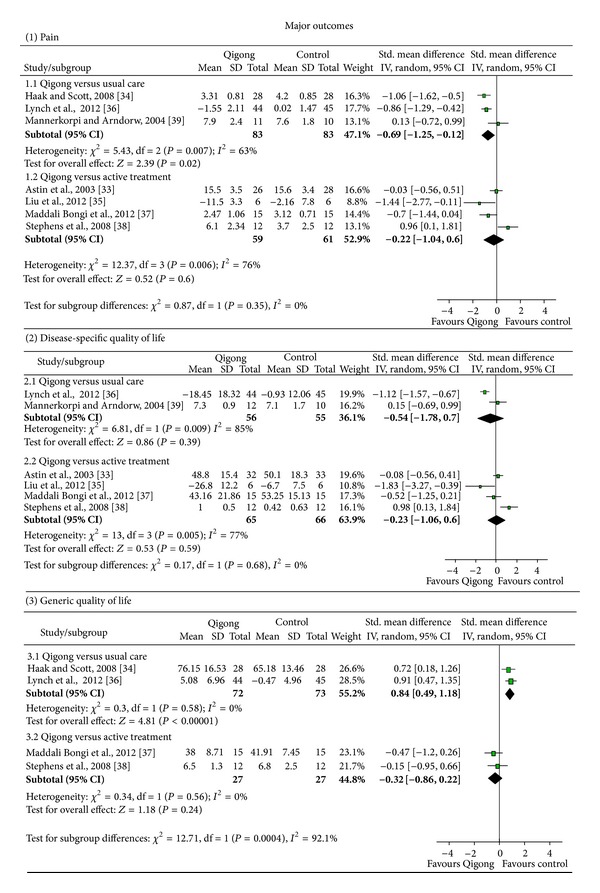
Forrest plots for major short-term outcomes.

1. Pain: evidence was found for a moderate short-term effect of Qigong on pain intensity (SMD = −0.69; 95% CI −1.25 to −0.12; *P* < 0.02; heterogeneity: *I* ^2^ = 63%; *χ* ^2^ = 5.43; *P* = 0.07) (Figure 2). Long-term effects could not be assessed because there was only one study [36], which showed a moderate effect on pain intensity (SMD = −0.51; 95% CI −0.93 to −0.08; *P* = 0.02).
2. Disease specific quality of life: no short-term effects were found for disease-specific quality of life (SMD = −0.54; 95% CI −1.78 to 0.70; *P* = 0.39) (Figure 2). Long-term effects could not be calculated; however, results of the only study [36] with that outcome indicated a strong effect (SMD = −1.10; 95% CI −1.55 to −0.65; *P* < 0.001).
3. Generic quality of life: a strong short-term effect was found for generic quality of life (SMD = 0.84; 95% CI 0.49 to 1.18; *P* < 0.001; heterogeneity: *I* ^2^ = 0%; *χ* ^2^ = 0.30; *P* = 0.58) (Figure 2). Only one study [36] investigated the long-term effects on generic quality of life with a moderate effect (SMD = 0.64; 95% CI 0.21 to 1.07; *P* = 0.003).

*Secondary Outcomes*

**Figure 3:**
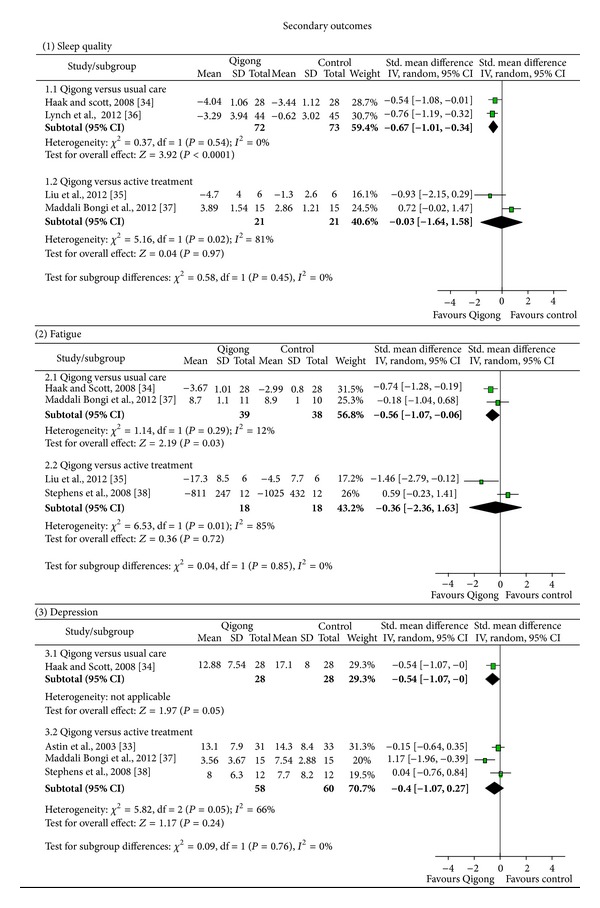
Forrest plots for secondary short-term outcomes.

1. Sleep quality: a moderate short-term effect of Qigong on sleep quality was found (SMD = −0.67; 95% CI −1.01 to −0.34; *P* < 0.001; heterogeneity: *I* ^2^ = 0%; *χ* ^2^ = 0.37; *P* = 0.54) (Figure 3). For long-term comparison, only data from one study [36] were available indicating a moderate long-term effect (SMD = −0.66; 95% CI −1.09 to −0.23; *P* = 0.003).
2. Fatigue: evidence was found for a moderate short-term effect on fatigue (SMD = −0.56; 95% CI −1.07 to −0.06; *P* = 0.03; heterogeneity: *I* ^2^ = 12%; *χ* ^2^ = 1.14; *P* = 0.29) (Figure 3). No data were available for long-term comparisons.
3. Depression: no effect could be calculated for short-term influences on depression. The only study available [34] indicated a significant effect on depression (SMD = −0.54; 95% CI −1.07 to −0.00; *P* < 0.05) (Figure 3). No data were available for long-term comparisons.

#### 3.3.2. Subgroup and Sensitivity Analyses of Qigong versus Waitlist/Usual Care

A subgroup analysis showed that, after exclusion, the study that used Qigong as part of a comprehensive program [39] and the significant effect on pain intensity remained in those who used Qigong as standalone treatment [34, 36]. For disease-specific quality of life, there was only one study left after exclusion [36]; therefore, it was not possible to draw reliable conclusions. For the other outcomes, no subgroup analyses were possible, because the study by Mannerkorpi and Arndorw [39] did not assess any other outcomes.

No sensitivity analyses could be conducted for low versus high risk of selection bias, since no study had high risk, and the same was true for detection bias and other bias. Analyses regarding attrition bias showed no changes on pain, generic quality of life, sleep quality, and depression after exclusion of the high risk study [39]. After exclusion only one study was left on fatigue, still with a significant effect (SMD = −0.74; 95% CI −1.28 to −0.19; *P* = 0.008). 

Sensitivity analyses for determination of causes for heterogeneity also revealed that the study by Mannerkorpi and Arndorw [39] was the main contributor for heterogeneity. Since that study was excluded in prior sensitivity analyses, no further analyses seemed necessary.

#### 3.3.3. Quality of Evidence

The quality of evidence according to GRADE was judged low for pain, quality of life, and sleep quality. Since only RCTs were included, the quality of evidence started at high quality, and then downgraded because of serious issues regarding risk of bias (−1) and imprecision due to small sample size (−1). For fatigue, the quality of evidence was judged very low after another downgrading was done because of inconsistency of the results (−1). 

### 3.4. Qigong versus Active Treatment

#### 3.4.1. Analyses of Effects of Qigong versus Active Treatment

*Major Outcomes*

1. Pain: no effects were found for short- (SMD = −0.22; 95% CI −1.04 to 0.60; *P* = 0.60) or long-term effects (SMD = −0.04; 95% CI −0.58 to 0.49; *P* = 0.87) of Qigong compared to active treatments on pain intensity (Figure 2).
2. Disease specific quality of life: no effect was found for disease-specific quality of life on the short-term (SMD = −0.23; 95% CI −1.06 to 0.60; *P* = 0.59) (Figure 2). No long-term effects could be assessed; the only available study [33] indicated no significant effect (SMD = −0.19; 95% CI −0.68 to 0.30; *P* = 0.45).
3. Generic quality of life: no effect was found for the short-term comparison of Qigong versus active treatment (SMD = −0.32; 95% CI −0.86 to 0.22; *P* = 0.24) (Figure 2). No study assessed long-term effects.

*Secondary Outcomes*

1. Sleep quality: no effect of Qigong was found for sleep quality on the short-term (SMD = −0.03; 95% CI −1.64 to 1.58; *P* = 0.97) (Figure 3). Long-term comparisons were not possible due to lack of data.
2. Fatigue: no effect of Qigong was found for fatigue on the short-term (SMD = −0.36; 95% CI −2.36 to 1.63; *P* = 0.72) (Figure 3). Long-term comparisons were not possible due to lack of data.
3. Depression: no effect was found for depression on the short-term (SMD = −0.40; 95% CI −1.07 to 0.27; *P* = 0.24) (Figure 3). No long-term effects could be assessed; the only available study [33] indicated no significant effect (SMD = −0.20; 95% CI −0.69 to 0.29; *P* = 0.43).

#### 3.4.2. Sensitivity Analyses of Qigong versus Active Treatment

No sensitivity analyses were conducted due to the absence of any significant effect.

### 3.5. Safety

Only three studies [36–38] reported adverse events. Two studies stated that no adverse events occurred [37, 38], and the third study [36] found two adverse events, namely, shoulder pain and plantar fasciitis; both events were transitory and fully resolved over time.

### 3.6. Risk of Bias across Studies

As less than ten studies were included in each meta-analysis, funnel plots were not analyzed.

### 3.7. Strength of Recommendation

Despite the fact that Qigong was not associated with serious adverse events, only weak recommendations could be made, mainly due to the small number of studies and low quality of evidence.

## 4. Discussion

### 4.1. Summary of Main Results

This meta-analysis found low quality evidence for moderate-to-strong effects of Qigong on pain, quality of life, and sleep quality and very low quality evidence for moderate effects on fatigue, when compared to usual care control groups. No effects were found for disease-specific quality of life and no effects could be calculated for depression or any long-term outcome. This meta-analysis also found no evidence for effects of Qigong on any outcome when compared to active control groups.

Data on safety were reported only in three studies with no occurrence of serious adverse events.

### 4.2. Applicability of Evidence

The reviewed trials were conducted in primary and secondary care settings in different countries. Most patients were adults in their 50s (except for 30 children [38]) and female; some studies did not state the numbers of male and female patients. All but one study [34] included patients with a diagnosis according to the ACR 1990 classification [40]. Cointerventions were mentioned in three studies [34, 35, 39] only and they consisted primarily of medication. Knowing that fibromyalgia patients are mainly female [1] and treated in primary and secondary care [6], this review's results potentially apply to the majority patients with fibromyalgia.

### 4.3. Quality of Evidence

Whilst the methodological quality of the studies reviewed differed somewhat, the effects of Qigong compared to usual care were robust against methodological bias. After excluding high risk studies, these effects remained for the most part. According to the GRADE recommendations, the quality of evidence ranged from low (for pain, quality of life, and sleep quality) to very low (for fatigue).

### 4.4. Agreements and Disagreements with Other Systematic Reviews

A thorough literature search located three other reviews of Qigong for fibromyalgia [7–9]; however, in one of them it was part of a larger review on exercise [9] or meditative movement therapies [7]. While the first review [9] included only two studies on Qigong for fibromyalgia, Langhorst et al. [7] assessed three studies in a meta-analysis [34, 38, 39] and concluded that they found no evidence to support its efficacy. The most recent review [8] reported on four RCTs, but did not conduct a meta-analysis and concluded that it was too early to judge the efficacy of Qigong for fibromyalgia. 

Given the seven RCTs included in this review and the evidence found for effects of Qigong compared to usual care but not to active controls, the review suggests that Qigong might be effective on the short term. The effect sizes are also mostly comparable to those of aerobic exercise [41]. Unfortunately this review and meta-analysis does not allow for conclusions regarding the long-term efficacy.

### 4.5. Strengths and Weaknesses

This review and meta-analysis study was conducted in accordance with the recommendations with the Cochrane Musculoskeletal Group [16] and for the first time reliable conclusions on the efficacy of Qigong could be drawn.

The review's primary limitation is the paucity of eligible trials, which rendered further subgroup analyses impossible. Studies especially comparing Qigong to other active therapies and studies investigating long-term effects are urgently needed.

Another limitation is the fact that in some studies Qigong was part of more comprehensive treatment program such as mindfulness meditation [33] or body awareness therapy [39], which makes it almost impossible to determine the isolated effect of Qigong.

Limiting factors are not only based on the studies design but also on the reporting of the conduction and the results. Most studies did not report randomization, allocation concealment, or blinding sufficiently. Some of the studies also used statistical within group comparisons but not between group comparisons despite the randomized controlled study design. Future studies should address these critical issues.

Heterogeneity was present in some meta-analyses; however, due to the small number of studies heterogeneity could not always be determined in sensitivity analyses.

### 4.6. Strength of Recommendation

According to GRADE, only a weak recommendation for Qigong can be made at this point.

## 5. Conclusion

This systematic review found low quality evidence for a short-term improvement of pain, quality of life, sleep quality, and very low quality evidence for improvement of fatigue after Qigong for fibromyalgia, when compared to usual care intervention. Given the low number of reported adverse events, Qigong may be a useful and safe approach in treating fibromyalgia. No evidence was found for Qigong compared to other active treatments. Further high quality RCTs that compare Qigong to established therapies (e.g., defined drug treatment, aerobic exercise) that report responder rates (e.g., −30% pain reduction) and that systematically assess adverse events are required for the conclusive judgment of its long-term effects.

## Figures and Tables

**Table 1:** Characteristics of the included studies.

Reference	Patients (*N*, diagnosis, age, gender)	Cointerventions	Intervention groups (*N*, contents, program length, frequency, duration)		Follow-up time points	Outcome measures	Results	
			Treatment	Control			Short-term	Long-term
						(1) Pain (2) Quality of life (disease specific, generic) (3) Sleep quality (4) Fatigue (5) Depression (6) Safety		

Astin et al., 2003 [33]	128 patients with fibromyalgia according to the ACR criteria Mean age: 47.7 ± 10.6 years Gender: 63/1 f/m (Qigong) 64/0 f/m (education)	Not reported	*Qigong* (*N* = 64) (mindfulness meditation and Qigong) 8 weeks once weekly 150 minutes each (90 min mindfulness meditation, 60 min Qigong)	*Education* (*N* = 64) (education and support group) 8 weeks once weekly 150 minutes each	8 weeks 24 weeks	(1) Pain intensity (Tender point tenderness) (2) Quality of life (FIQ) (5) Depression (BDI)	(1) Sign. improvement in Qigong (2) Sign. improvement in Qigong and Education (5) Sign. improvement in Qigong and education	(1) Sign. improvement in Qigong. (2) Sign. improvement in Qigong and education (5) Sign. improvement in Qigong and education

Haak and Scott, 2008 [34]	57 female patients with fibromyalgia (diagnosis unclear) Mean age: 53.3 ± 9.2 years Gender: female only	Medication	*Qigong* (*N* = 29) (He Hua Qigong-Lotus method) 7 weeks 9 sessions 11.5 hours in total + home practice twice weekly 20 min each +2 sessions of external Qigong from Qigong master	*Usual care* (*N* = 28) 7 weeks	End of intervention (not specified) 4 months (combined group, all patients had received the intervention)	(1) Pain intensity (VNS) (2) Quality of life (WHOQOL-BREF) (3) Sleep quality (VNS) (5) Depression (BDI)	(1) Sign. differences favoring Qigong (2) Sign. differences favoring Qigong (3) No sign. group difference (5) Sign. differences favoring Qigong	(1) Sign. improvement compared to baseline (2) Sign. improvement compared to baseline (3) No change (5) Sign. improvement compared to baseline

Liu et al., 2012 [35]	14 patients with fibromyalgia according to the ACR criteria Mean age: 55.7 years (Qigong) 57.7 years (Sham Qigong) Gender: female only	Medication (stable dosage, for pain, sleep, or mild depression)	*Qigong* (*N* = 8) (Liu Zi Jue Qigong—“six healing sounds” Qigong: standing, sitting, supine postures, breathing exercises, quiet chanting, meditation) 2 initial sessions 45–60 min each + 6 weeks once weekly 45–60 min each + home practice twice daily 15–20 min each	*Sham Qigong* (*N* = 6) (matched standing, sitting, supine postures) 2 initial sessions 45–60 min each + 6 weeks once weekly 45–60 min each + home practice twice daily 15–20 min each	End of intervention (not specified)	(1) Pain intensity (SMPQ) (2) Quality of life (FIQ) (3) Sleep quality (PSQI) (4) Fatigue (MFI)	(1) Sign. differences favoring Qigong (2) No sign. group difference (3) Sign. differences favoring Qigong (4) Sign. differences favoring Qigong	

Lynch et al., 2012 [36]	100 patients with fibromyalgia according to the ACR criteria Mean age: 52 years Gender: 50/3 f/m (Qigong) 46/1 f/m (usual care)	Not reported	*Qigong* (*N* = 53) (Chaoqi Fanhuan Qigong) 3 consecutive days +8 weeks once weekly 60 min each +6 months home practice daily 45–60 min each	*Usual care* (*N* = 47)	6 months	(1) Pain intensity (NRS) (2) Quality of life (FIQ, SF-36) (3) Sleep quality (PSQI) (6) Adverse events	(1) Sign. differences favoring Qigong (2) FIQ: Sign. differences favoring Qigong SF-36: Sign. differences favoring Qigong (3) Sign. differences favoring Qigong (6) 2 adverse events (shoulder pain, plantar fasciitis)	(1) Sign. differences favoring Qigong (2) FIQ: Sign. differences favoring Qigong SF-36: Sign. differences favoring Qigong (3) Sign. differences favoring Qigong

Maddali Bongi et al., 2012 [37]	30 patients with fibromyalgia according to the ACR criteria Mean age: 56.6 ± 9.10 (Qigong) 57.9 ± 13.5 (Rességuier Method) Gender: not reported	Not reported	*Qigong* (*N* = 15) (static and dynamic exercises, breathing exercises, self-massage) 3 weeks twice weekly 45 min each +4 weeks once weekly 45 min each	*Rességuier Method* (*N* = 15) (training of awareness and control of bodily perceptions) 3 weeks twice weekly 60 min each +4 weeks once weekly 60 min each +home practise once daily 30 min each	7 weeks	(1) Pain (NRS) (2) Quality of life (FIQ, SF-36) (3) Sleep quality (NRS) (5) Depression (HADS) (6) Adverse events	(1) Sign. improvement in Qigong and Rességuier method (2) FIQ: Sign. in Qigong and Rességuier method SF-36: Sign. in Qigong and Rességuier method (3) No sign. change (5) Sign. improvement in Qigong (6) No adverse events	

Mannerkorpi and Arndorw, 2004 [39]	36 female patients with fibromyalgia according to the ACR criteria Mean age: 45 ± 8.3 years Gender: female only	Medication (analgesics, antidepressants, sedatives)	*Qigong* (*N* = 19) (body awareness therapy and Qigong) 3 months once weekly 90 min each (incl. 20 min Qigong)	*Usual care* (*N* = 17)	End of intervention (not specified)	(2) Quality of life (FIQ)	(4) No sign. group difference	

Stephens et al., 2008 [38]	30 children (8–18 years) with fibromyalgia according to the ACR criteria Mean age: 12.9 ± 2.7 years (Qigong) 13.6 ± 1.84 years (Exercise) Gender: 12/4 f/m (Qigong) 10/4 f/m (education)	Not reported	*Qigong* (*N* = 16) (postures, relaxation) 12 weeks three times weekly 35 min each	*Exercise* (*N* = 14) (Aerobic exercise: cardiodance, boxing movements, stretching) 12 weeks three times weekly 50 min each	End of intervention (not specified)	(1) Pain intensity (PedsQL) (2) Quality of life (C-HAQ, QOML) (4) Fatigue (PedsQL) (5) Depression (CDI) (6) Adverse events	(1) No sign. group difference (2) C-HAQ: sign. differences favoring exercise QOML: sign. differences favoring exercise (4) Sign. differences favoring exercise (5) No sign. group difference (6) No adverse events	

Abbreviations: ACR: American College of Rheumatology; BDI: Beck Depression Inventory; CDI: Childhood Depression Inventory; C-HAQ: Childhood Health Assessment Questionnaire; f: female; FIQ: Fibromyalgia Impact Questionnaire; HADS: Hospital Anxiety and Depression Scale; m: male; MFI: Multidimensional Fatigue Inventory; NRS: Numerical Rating Scale; PedsQL: Pediatric Quality of Life Inventory; PSQI: Pittsburgh Sleep Quality Index; QOML: Quality of My Life; SF-36: Short Form 36 Health Survey; Sign.: significant; SMPQ: Short-Form McGill Pain Questionnaire; VNS: Visual Numerical Scale; WHOQOL-BREF: World Health Organization Quality of Life BREF.

**Table 2:** Risk of bias summary: review authors' judgments about each risk of bias item for each included study.

	Random sequence generation	Allocation concealment	Blinding of participants and personnel	Blinding of outcome assessment	Incomplete outcome data	Selective reporting	Other bias
	(selection bias)	(selection bias)	(performance bias)	(detection bias)	(attrition bias)	(reporting bias)	
Astin et al., 2003 [33]	+	+	?	+	−	+	+
Haak and Scott,2008 [34]	?	?	?	+	+	−	+
Liu et al., 2012 [35]	?	?	−	+	+	+	+
Lynch et al., 2012 [36]	+	?	?	+	+	+	+
Maddali Bongi et al., 2012 [37]	+	+	?	+	+	+	+
Mannerkorpi and Arndorw,2004 [39]	?	?	?	+	−	+	+
Stephens et al., 2008 [38]	+	+	?	+	−	+	+

“+” means low risk, “−” means high risk, and “?” means unclear risk of bias.
